# Transient restrictive cardiomyopathy in cats: first reported case series

**DOI:** 10.3389/fvets.2025.1607384

**Published:** 2025-05-23

**Authors:** Camille Poissonnier, Pierre Guigo, Pierre Foulex, Peggy Passavin, Aurore Fouhety, Tiphaine Douay, Coline Le Gall, Kahina Kartout, Éric Bomassi, Valérie Chetboul

**Affiliations:** ^1^Université Paris-Est, École Nationale Vétérinaire d’Alfort, ChuvAc, Maisons-Alfort, France; ^2^Clinique Vétérinaire Tourainevet, Rochecorbon, France; ^3^Clinique Vétérinaire Boulogne Roland Garros, Boulogne Billancourt, France; ^4^Centre Hospitalier Vétérinaire Atlantia, Nantes, France; ^5^Clinique vétérinaire EVOLIA, IVC Evidensia France, l'Isle Adam, France; ^6^Clinique vétérinaire Olliolis, Ollioules, France; ^7^U955 - IMRB Inserm, École Nationale Vétérinaire d’Alfort, UPEC, Maisons-Alfort, France

**Keywords:** cardiology, echocardiography, feline, myocardium, troponin I

## Abstract

**Background:**

Transient myocardial thickening has been reported in cats. This clinical entity is characterized by reversible left ventricular (LV) hypertrophy often associated with left atrial (LA) dilation and congestive heart failure (CHF) that resolves over time. To the best of our knowledge, transient restrictive cardiomyopathy (TRCM) has not yet been reported in cats.

**Objectives:**

To describe the epidemiological, clinical, echocardiographic, and prognostic features of cats with TRCM.

**Animals:**

17 cats with TRCM and 16 control cats with non-transient RCM (NT-RCM).

**Methods:**

Retrospective study. Clinical records of cats with an RCM phenotype (defined by normal LV dimensions with LA or biatrial enlargement) were searched in 6 veterinary databases. Cats with subsequent resolution of the RCM phenotype were included in the TRCM group and those with persistent RCM phenotype in the NT-RCM group.

**Results:**

TRCM cats were significantly younger than NT-RCM cats (*p* = 0.003). An antecedent event was identified 24 h to 17 days before diagnosis in most TRCM cats (11/17), but in no NT-RCM cat. All TRCM cats and 12/16 NT-RCM cats had CHF, with a lower left atrium:aorta ratio in TRCM cats (*p* = 0.04). Diuretic treatment was discontinued (10/17) or decreased (7/17) in all TRCM cats, but in no NT-RCM cat. Median survival time of NT-RCM cats was 667 days whereas the Kaplan–Meier method estimated that 86% of the TRCM cats had not died from cardiac death 6,000 days after diagnosis (*p* = 0.003).

**Conclusion and clinical importance:**

TRCM occurs in cats with common antecedent events and with excellent long-term prognosis in most cases.

## 1 Introduction

Cardiomyopathies refer to a broad spectrum of heterogeneous diseases defined as myocardial disorders in which the heart muscle is structurally and functionally abnormal in the absence of any other cardiovascular disease sufficient to cause the observed myocardial abnormality (1, 2). Cardiomyopathies are the most common feline heart diseases characterized by various structural and functional characteristics also called phenotypes, with four different clinical stages (from A to D) and a variable prognosis (1, 2). In 2020, the American College of Veterinary Internal Medicine (ACVIM) consensus statement panel on feline cardiomyopathies proposed a classification scheme including five phenotypic categories diagnosed by echocardiography, i.e., the hypertrophic cardiomyopathy (HCM), restrictive cardiomyopathy (RCM), dilated cardiomyopathy, arrhythmogenic right ventricular cardiomyopathy, and nonspecific cardiomyopathy phenotypes (1). The HCM phenotype characterized by increased diffuse or regional left ventricular (LV) wall thickness with a non-dilated LV chamber is predominant in cats, with an estimated global prevalence of 15% increasing with age (3). The most common cause of HCM phenotype is primary HCM, also called HCM, other causes including systemic hypertension, hyperthyroidism, acromegaly, neoplastic infiltration, reduced preload, and myocarditis (1, 2). A particular cause of feline HCM phenotype reported as “transient myocardial thickening” (TMT) has been recently described, characterized by reversible LV wall thickening which mimics HCM at initiation (4, 5). The exact mechanisms and causes of this pathological condition are currently unknown, although a transient interstitial edema due to myocarditis is highly suspected, as reported in humans (6, 7), with a particular antecedent event often identified as possible cause, such as general anesthesia for various procedures (e.g., neutering, tooth extraction), road traffic accidents, pneumonia, bite wound, abdominal pain, vaccination, toxoplamosis, bartonellosis, and fever of unknown origin (4, 5, 8, 9).

The RCM phenotype, defined in its myocardial form by normal LV dimensions with left atrial (LA) or biatrial enlargement, is considered as the second most common feline cardiomyopathy phenotype (1, 2, 10–15). Primary RCM and less commonly hyperthyroidism are the two causes of the RCM phenotype (1, 13, 14). To the best of our knowledge, transient restrictive cardiomyopathy (TRCM) has not yet been reported in cats. However, the authors of the present study had noticed that some cats with an RCM phenotype appeared to show decrease in LA size over time, and then disappearance of LA or biatrial enlargement with a good to excellent prognosis similar to that reported for feline TMT (4, 5, 15).

The aim of the present multicenter retrospective study was therefore to search for these particular TRCM cases and to describe their historical, epidemiological, clinical, diagnostic, and outcome features.

## 2 Materials and methods

### 2.1 Animals

The medical and echocardiographic database of 6 cardiology referral centers from 2005 to 2024 (Tourainevet, Clinique Vétérinaire Boulogne Roland Garros, Alfort Veterinary School, Atlantia, Evolia, Olliolis) were retrospectively reviewed to search for cats diagnosed with RCM and for which follow-up revealed either disappearance or persistence of the RCM phenotype over time. All cases were reviewed by a board-certified specialist (Dipl.ECVIM-CA cardiology, VC). All included cats had therefore at least two echocardiographic examinations (Vivid iq, 7, E9, E95 General Electric medical system, Waukesha, WI, United States; Megas and MyLab Twice, Esaote Biomedica, Firenze, Italy). All selected cats were characterized at inclusion by a typical RCM phenotype in its myocardial form, as defined by the ACVIM consensus statement on feline cardiomyopathies, i.e., normal LV dimensions with LA or biatrial enlargement on echocardiographic examination (1). Cats were included in the TRCM group if follow-up confirmed resolution of CHF (when present at inclusion), along with disappearance of the initial RCM phenotype on a second echocardiographic examination, i.e., normalization of LA or biatrial size with persistent normal LV dimensions. A control group of cats with non-transient RCM (NT-RCM) was also recruited from the same databases. Cats were included in the NT-RCM group if a second echocardiographic examination performed 1 to 3 months after the initial diagnosis confirmed worsening of the RCM phenotype or if a second echocardiographic examination performed > 3 months after the initial diagnosis confirmed either the worsening or persistence of the RCM phenotype.

The main epidemiological (age, body weight, sex, and breed) and clinical characteristics of the included animals were extracted from computerized databases. The medical history and more particularly the presence of an antecedent event prior to presentation was searched (i.e., an unusual event occurring in the life of the cat as described in TMT cases) (4, 5). In the case of respiratory signs (e.g., respiratory distress), thoracic radiographs (lateral and dorsoventral views) were obtained to search for signs consistent with congestive heart failure (CHF), defined as the concurrent presence of compatible clinical signs (i.e., respiratory distress) and thoracic radiographic abnormalites (i.e., interstitial or mixed interstitial and alveolar pattern and/or pleural effusion). Thoracic radiographs findings regarding CHF status were also recorded.

Cats suffering from endomyocardial form of RCM, from other types of cardiomyopathy phenotypes, cats with systolic anterior motion of the mitral valve or for which the cardiac diagnosis was equivocal were not included in the study. The presence of a systemic disease known to affect the cardiovascular health was also considered as a non-inclusion criterion (e.g., hyperthyroidism and systemic arterial hypertension).

### 2.2 Echocardiographic data

Standard transthoracic M-mode, two-dimensional (2D) mode, and Doppler examinations were performed by trained observers in awake cats and all echocardiographic data were reviewed by an ECVIM-CA board-certified veterinary cardiologist (VC).

#### 2.2.1 Two-dimensional and M-mode measurements

Left ventricular end-diastolic and end-systolic internal diameters (LVDd and LVDs respectively), LV free wall thicknesses in diastole (LVFWd) and systole (LVFWs), IVS thicknesses in diastole (IVSd) and systole (IVSs) were measured using the 2D-guided M-mode using the leading edge-to-leading edge method, and the LV shortening fraction (SF%) was then calculated (1). The subaortic interventricular septal thickness was also measured at end-diastole by 2D mode from the right parasternal 5-chamber view at the mitral valve-chordae tendineae interface and compared with reference ranges, to exclude sub-aortic focal myocardial hypertrophy (16).

For each cat, all M-mode measurements (IVSd, IVSs, LVFWd, LVFWs, LVDd, LVDs, and SF%) were compared to the 95% prediction intervals assessed according to body weight from a large population of healthy cats (17).

The LA and aortic (Ao) diameters were measured at end-diastole by a 2D method from the right parasternal short axis view, as previously described (15, 16). The LA:Ao ratio was then calculated, and LA enlargement was defined as an LA:Ao ratio > 1.2 (upper cut-off value obtained from a population of 100 prospectively recruited healthy cats (16)). Additionally, the maximum LA diameter (Max LAD) was measured at end-systole from the interatrial septum to the LA free wall using the right parasternal 4-chamber view, and LA enlargement was confirmed for Max LAD values > 16 mm (18). Lastly, the right end-diastolic atrial diameter was also measured at the level of the tricuspid annulus from the 2D right parasternal 4-chamber view, and the right atrium was considered dilated if its diameter was greater than the upper reference limit (> 15 mm) obtained from a population of 120 healthy adult cats (15).

#### 2.2.2 Conventional Doppler examination

Pulsed-wave and continuous-wave Doppler modes were used to record blood flow velocities. Maximum early (E) and late (A) diastolic mitral flow velocities were measured when possible (i.e., non-merged E and A waves) using pulsed-wave Doppler mode from the left apical 4-chamber view, and the mitral E:A ratio was then calculated. Continuous-wave Doppler recorded from the left apical 5-chamber view was used to exclude LV outflow tract obstruction defined by both turbulent aortic flow of high velocity (> 2 m/s) (16) and the presence of systolic anterior motion of the mitral valve (which was checked using 2D right parasternal long-axis images and M-mode mitral valve images obtained from right parasternal short- and long-axis image planes). Color-flow and spectral Doppler modes were also used to identify potential valve regurgitations and exclude shunting and stenotic lesions.

### 2.3 Outcome data

Follow-up was carefully analyzed for all included cats. Data from the last examination were recorded and compared to the initial examination for each cat in both groups.

Owners of cats for which the outcome could not be found in the database at the time of writing were contacted by telephone or e-mail to determine the current status of their animals: alive or dead (date and cause of death, defined as cardiac or non-cardiac). Cases of animals dying suddenly, euthanized for cardiac reasons or dying from nonresponsive CHF or arterial thromboembolism were considered as cardiac deaths. Sudden deaths included sudden witnessed deaths and suspected sudden deaths (cats found dead at home without an obvious cause and completely asymptomatic during the previous 24 h). Cats for which the outcome could not be obtained at the time of writing were considered lost to follow-up and were consequently censored at the time of their last examination.

### 2.4 Statistical analysis

Data are described as median [interquartile range, IQR; minimum-maximum]. Statistical analysis was performed by a commercially available software [DATAtab Team (2024). DATAtab: Online Statistics Calculator. DATAtab e.U. Graz, Austria]. Mann–Whitney Wilcoxon and Fisher tests were performed to compare TRCM cats with NT-RCM cats. Median survival times to all-cause death and to cardiac death were both estimated using the Kaplan–Meier method and compared by applying a log rank test. The level of significance was set at *p* < 0.05.

## 3 Results

### 3.1 Study population

A total of 33 cats with an RCM phenotype were included in the study, i.e., 17 cats with TRCM and 16 control cats with NT-RCM. Epidemiological and clinical features of the study population are presented in Table 1. Cats with TRCM were significantly younger than NT-RCM cats [median age (IQR; minimum-maximum) of 3.4 years (0.7–10.6 years; 0.4–14.2 years) and 10.8 years (9.1–12.3 years; 6.3–20.3 years) respectively; *p* = 0.003]. Sex distribution was not different between the two groups (*p* = 0.08).

**Table 1:** Selected epidemiological and clinical features of cats with transient restrictive cardiomyopathy phenotype (TRCM group; *n* = 17) at initial presentation as compared to a control contemporary population of 16 cats with non-transient restrictive cardiomyopathy phenotype (NT-RCM group).

Characteristic		TRCM group (*n* = 17)	NT-RCM group (*n* = 16)	**P**
Sex	Male	35% (6/17)	69% (11/16)	0.08
	Female	65% (11/17)	31% (5/16)	
Age (years) median [IQR]		3.4 [0.7–10.6]	10.8 [9.1–12.3]	**0.003**
Body weight (kg) median [IQR]		3.8 [3.5–5.0]	4.3 [3.4–5.8]	0.60
Feline breeds	Domestic Shorthair	82% (14/17)	81% (13/16)	
	British Shorthair	6% (1/17)	0% (0/16)	
	Persian	6% (1/17)	0% (0/16)	
	Siamese	6% (1/17)	6% (1/16)	
	Sphynx	0% (0/17)	6% (1/16)	
	Turkish Angora	0% (0/17)	6% (1/16)	
Congestive heart failure (CHF)	CHF: yes (number, %)	100% (17/17)	75% (12/16)	**0.04**
	Pulmonary edema	71% (12/17)	19% (3/16)	**0.005**
	Pleural effusion	53% (9/17)	69% (11/16)	0.48
	Pericardial effusion	12% (2/17)	19% (3/16)	1.0
	Ascites	0% (0/17)	13% (2/16)	0.23
	Dyspnea	82% (14/17)	69% (11/16)	0.43
	Weakness	12% (2/17)	19% (3/16)	0.66
Clinical signs	Weight loss	12% (2/17)	25% (4/16)	0.40
	Syncope	0% (0/17)	13% (2/16)	0.48
	Abdominal distension (ascites)	0% (0/17)	13% (2/16)	0.23

Bold values indicate statistically significant *p* values.

Antecedent events were identified 24 h to 17 days (median = 7 days) in 11/17 (65%) cats with TRCM, i.e., neutering (*n* = 5), respiratory diseases (rhinitis and bronchopneumonia, *n* = 2), a single glucocorticoid injection for feline eosinophilic granuloma treatment 2 weeks previous to the presentation for CHF (*n* = 1), gastrointestinal signs in a cat with combined infection with feline leukemia virus and feline immunodeficiency virus (*n* = 1), pyelonephritis (*n* = 1), and urethrostomy to treat feline lower urinary tract disease (*n* = 1). Conversely, no antecedent event was reported for any of the NT-RCM cats. A complete blood count was performed in 4/17 TRCM cats and identified eosinophilia in one cat, and neutrophilic leukocytosis in three cats including the one with bronchopneumonia. Clinical signs (prevalence and type) were not different among the two groups (Table 1).

Thoracic radiographs and echocardiography confirmed CHF in all TRCM cats and in most (75%) NT-RCM cats (Table 1). Pulmonary edema was more frequently observed in TRCM cats than NT-RCM cats (*p* = 0.005). Other types of CHF (i.e., pleural effusion, pericardial effusion, ascites) were not significantly different between the two feline groups (*p* = 0.48, *p* = 1.0, *p* = 0.23, respectively).

### 3.2 Echocardiographic findings at inclusion

Echocardiographic data of the study population are presented in Table 2. According to the chosen inclusion criteria, all included cats showed normal LV dimensions as compared with 95% prediction intervals assessed according to body weight from a large population of healthy cats (17), with LA dilation or biatrial enlargement detected in, respectively, 9/17 (53%) and 8/17 (47%) TRCM cats. More than two third of the cats from the NT-RCM group (11/16, 69%) had biatrial enlargement, and the remaining cats (5/16, 31%) LA enlargement only. The proportion of cats with biatrial enlargement was not significantly different between the two groups (*p* = 0.30). The end-diastolic LA:Ao ratio at diagnosis was significantly lower (*p* = 0.04) in the TRCM group (1.80 [1.56–1.89; 1.33–2.30]) than in the NT-RCM group (2.02 [1.71–2.41;1.36–2.93]). Similarly, Max LAD at diagnosis was significantly lower (*p* = 0.001) in the TRCM group (18.1 mm [17.2–19.2; 16.8–20.2]) than in the NT-RCM group (21.0 mm [20.1–22.0; 19.5–24.1]). Conversely, SF% was significantly higher (*p* = 0.045) in the TRCM group (51 [45–62; 36–68]) than in the NT-RCM group (45 [33–48; 31–61]). All other 2D and M-mode echocardiographic variables evaluated at inclusion (IVSd, IVSs, LVFWd, LVFWs, LVDd, LVDs, RA diameter and the end-diastolic subaortic interventricular septal thickness) were not significantly different between the two groups. Additionally, none of the cats in either group had atrial thrombi, and there was no significant difference in the proportion of cats with spontaneous echo contrast: all TRCM cats (17/17) and all but one NT-RCM cat showed spontaneous echo contrast (15/16; *p* = 0.48).

**Table 2:** Selected echocardiographic and Doppler variables in cats with transient restrictive cardiomyopathy phenotype (n = 17; TRCM group) at initial presentation (Day 0) and at the final echocardiographic examination as compared to a control contemporary population of 16 cats without resolution of the restrictive cardiomyopathy phenotype (NT-RCM group).

Variables	TRCM group (*n* = 17)						NT-TRCM group (*n* = 16)						Comparisons between TRCM and NT-RCM groups	
	Values at day 0			Values at the final examination			Values at day 0			Values at the final examination			Day 0	Final examination
	n	Median	IQR	n	Median	IQR	n	Median	IQR	n	Median	IQR	*p*	*p*
M-mode echocardiographic variables														
IVSd (mm)	16	4.4	3.6–4.8	17	3.7	3.6–4.5	16	4.5	4.0–4.7	16	4.0	3.6–4.7	0.54	0.77
LVDd (mm)	16	16.0	14.3–18.2	17	14.3	13.5–15.3	16	15.5	14.9–18.2	16	17.8	15.8–19.2	1.0	**0.004**
LVFWd (mm)	16	4.5	3.7–4.7	17	3.9	3.6–4.4	16	4.0	4.0–4.5	16	4.0	3.2–4.3	0.56	0.50
IVSs (mm)	16	7.0	5.4–7.9	17	6.5	6.0–7.5	16	6.9	6.2–7.2	16	7.0	6.0–7.7	0.92	0.86
LVDs (mm)	16	7.4	5.9–9.5	17	7.3	6.8–7.8	16	9.1	7.9–10.6	16	8.5	7.1–10.0	0.06	0.09
LVFW (mm)	16	7.8	6.3–8.3	17	6.6	6.3–7.5	16	7.0	6.7–8.1	16	7.5	6.1–8.9	0.91	0.43
SF%	16	51	45–62	17	46	44–54	16	45	33–48	16	48	41–58	**0.045**	0.81
Two-dimensional echocardiographic variables														
SA-IVSd (mm)	14	4.3	4.1–4.7	14	3.8	3.4–4.1	12	4.45	4.00–4.73	9	4.3	4.0–5.0	0.76	0.12
End-diastolic LA:Ao ratio	17	1.80	1.56–1.89	17	0.97^b^	0.89–1.06	16	2.02	1.71–2.41	16	2.01	1.63–2.58	**0.04**	**<0.001**
Maximal LA diameter (mm)	6	18.1	17.2–19.2	9	12.0^b^	10.4–14.5	5	21.0	20.1–22.0	5	23.4	21.7–23.8	**0.001**	**0.003**
Right atrial diameter (mm)	16	14.1	9.7–15.4	16	10.7^a^	9.6–11.7	15	16.0	13.2–17.8	15	16.7	13.5–18.1	0.1	**<0.001**
Conventional Doppler variable														
Mitral E:A ratio	9	3.33	2.50–3.53	10	1.25^b^	1.15–1.60	6	3.68	2.63–3.70	7	3.00	2.98–4.24	0.59	**<0.001**

Ao, aorta. EMF, endomyocardial fibrosis. IQR, interquartile range. IVSd, end-diastolic interventricular septal thickness. IVSs, end-systolic interventricular septal thickness. LA, left atrium. LVd, end-diastolic left ventricular internal diameter. LVs, end-systolic left ventricular internal diameter. LVFWd, end-diastolic left ventricular free wall thickness. LVFWs, end-systolic left ventricular free wall thickness. RA, end-diastolic right atrial diameter. SA-IVSd, sub-aortic interventricular septal thickness measured at end-diastole. SF%: shortening fraction. Bold values indicate statistically significant *p* values for comparisons between TRCM and NT-RCM groups. In the NT-RCM group, echocardiographic and Doppler variables at Day 0 and at the final examination were not significantly different.

a Significantly (*p* < 0.05) different from value at Day 0 within the TRCM group.

b Significantly (*p* < 0.001) different from value at Day 0 within the TRCM group.

Lastly, the mitral E:A ratio could be measured in 9/17 TRCM cats and 6/16 NT-RCM cats at inclusion confirming a restrictive mitral inflow Doppler pattern for all cats (mitral E:A ratio > 2). No significant difference regarding mitral E:A ratio was observed between the two groups (3.33 [2.50–3.53; 2.20–4.00] and 3.68 [2.63–3.70; 2.35–3.81], respectively; *p* = 0.59).

### 3.3 Follow-up data and survival

**Figure 1:**
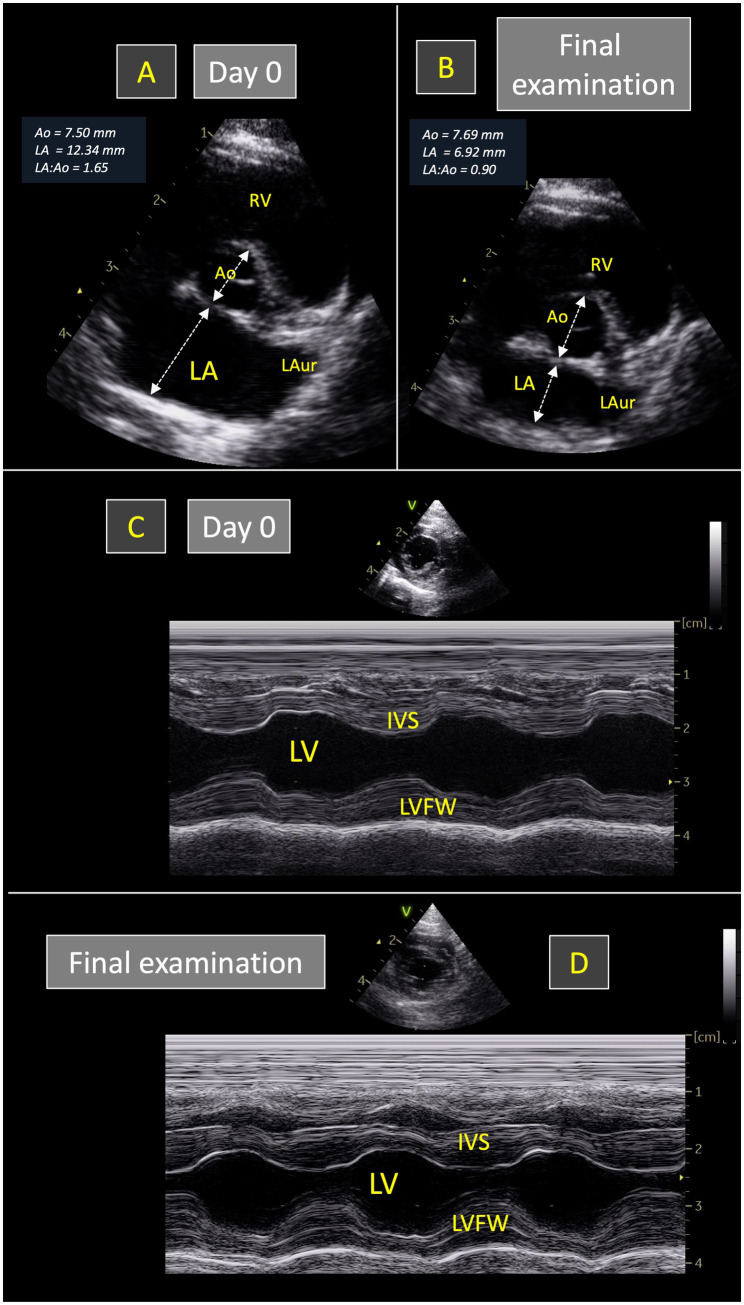
Representative echocardiograms recorded in one 3.5-kg cat with transient restrictive cardiomyopathy phenotype (from the TRCM group of the present study) at initial presentation (Day 0) and at final examination (35 days later). Short-axis right sided parasternal views obtained at the level of the aortic valve at end-diastole **(A,B)** show that the left atrium-to-aorta ratio (LA:Ao) was increased at Day 0 (1.65; **A**) and normalized at the final examination (0.90; **B**). As shown in **(C,D)**, left ventricular M-mode measurements remained within normal ranges at both examinations. IVS, interventricular septum. LAur, left auricle. LV, left ventricle. LVFW, left ventricular free wall. RV, right ventricle.

In the TRCM group, the median time from the RCM phenotype diagnosis to evidence of its echocardiographic resolution was 156 days [77–211 days; 14–453 days], with normalization of LA dimensions for all cats, as confirmed by both end-diastolic LA:Ao ratio and Max LAD within normal ranges (Table 2). The final median end-diastolic LA:Ao ratio of TRCM cats was 0.97 (0.89–1.06; 0.77–1.14) vs. 2.01 (1.63–2.58; 1.37–3.0) in the NT-RCM group (*p* < 0.001, Figure 1), and the final median Max LAD was 12.0 mm (10.4–14.5 mm; 9.9–15.0 mm) vs. 23.4 mm (21.7–23.8 mm; 21.6–24.9 mm) in the NT-RCM group (*p* = 0.003). Similarly, the right atrial dilation initially identified in 8/17 TRCM and 11/16 NT-RCM cats disappeared in all TRCM cats, while it remained dilated in all NT-RCM cats (Table 2). Additionally, the mitral E:A ratio normalized in all TRCM cats (1.25 [1.15–1.60; 0.79–1.95]), which was not the case for NT-RCM cats (3.0 [2.98–4.24; 2.28–6.0]; *p* < 0.001). Lastly, LV thicknesses and LV diameters remained within normal ranges for all TRCM and NT-RCM cats at the final examination and was not significatively different within and between each group.

Following the diagnosis of RCM phenotype, all cats with CHF (17/17 cats with TRCM and 12/16 cats with NT-RCM) were prescribed loop diuretics (furosemide or torsemide). Diuretic treatment was discontinued (10/17) or decreased (7/17) in all TRCM cats but in none of the NT-RCM cats. Additional cardiac treatment prescribed was clopidogrel (6/17 cats with TRCM, 11/16 cats with NT-RCM), pimobendane (4/17 cats with TRCM, 8/16 cats with NT-RCM), benazepril (6/17 cats with TRCM, 6/16 cats with NT-RCM), rivaroxaban (1/17 cats with TRCM, 2/16 cats with NT-RCM), and aspirin (2/17 cats with TRCM, 1/16 cats with NT-RCM).

Serum ultrasensitive cardiac troponin I was available in two TRCM cats. Values were markedly increased at diagnosis (18.3 and 4.3 ng/mL; reference range < 0.06 ng/mL) and dramatically decreased for both cats with normal and subnormal values 121 and 10 days after diagnosis (0.01 and 0.06 ng/mL, respectively).

**Figure 2:**
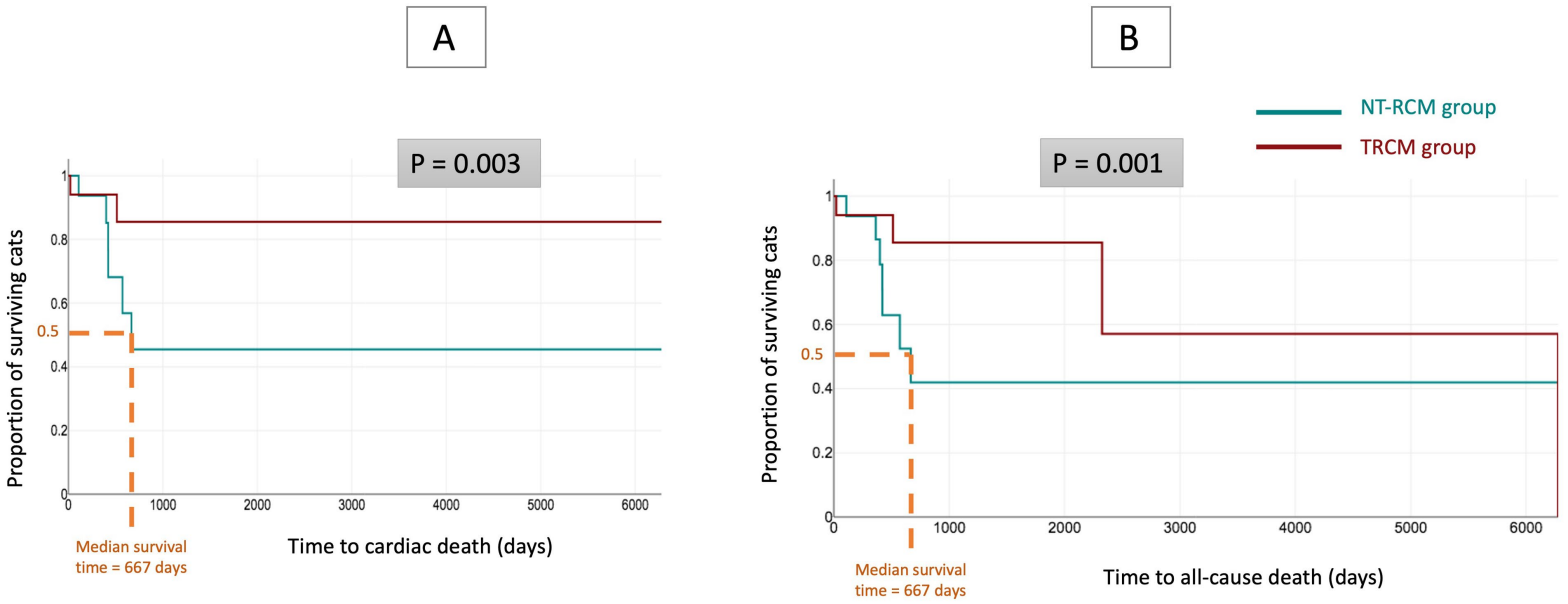
Kaplan–Meier curves illustrating survival time from initial diagnosis to cardiac death **(A)** and all-cause **(B)** death for cats from the transient restrictive cardiomyopathy group (TRCM group; *n* = 17), as compared with cats from the non-transient RCM group (NT-RCM group; *n* = 16). Considering both cardiac death and all-cause death, survival time was significantly different between the two groups (*p* = 0.003 and *p* = 0.001, respectively). Median survival time of NT-RCM cats was 667 days (95% confidence interval = 420–667 days for cardiac death and 421–667 days for all-cause death), whereas the Kaplan–Meier method estimated that 86 and 57% of TRCM cats had not died from cardiac death and all-cause death 6,000 days after diagnosis.

Considering both cardiac and all-cause mortality, 7 NT-RCM cats (6 from cardiac causes, 1 from non-cardiac causes) and 4 TRCM cats (2 from cardiac causes, 2 from non-cardiac causes) died during the follow-up period. Of the two TRCM cats that died from cardiac causes, one was euthanized for CHF due to an HCM phenotype secondary to systemic arterial hypertension and chronic kidney disease and the other died from unresolved ATE, respectively 481 and 2 days after RCM phenotype resolution. Median follow up time was 368 days (IQR = 104–424) for NT-RCM cats and 562 days (422–1,351) in the TRCM group. Median survival time was significantly different between the two feline groups (667 days in the NT-RCM group for cardiac death and also all-cause death vs. median survival time not reached in the TRCM group, *p* = 0.003 and *p* = 0.001 for time to cardiac death and all-cause death, respectively; Figure 2). The Kaplan–Meier method estimated that 86 and 57% of TRCM cats had not died from cardiac death and all-cause death 6,000 days after diagnosis.

## 4 Discussion

The present study demonstrates that another form of transient myocardial phenotype, i.e., TRCM, can be observed in cats with an excellent prognosis in most cases, similarly to feline TMT (4, 5). These two clinical entities that progressively resolve during follow-up are often preceded by a specific event that mainly occurs in the prior 2 weeks. Although the underlying pathophysiology of TRCM is currently unknown, its reversion suggests acute or subacute myocarditis as a probable origin, as suspected in TMT case series (4, 5, 9).

In cats, the RCM phenotype in its myocardial form is characterized by diastolic dysfunction secondary to severe diffuse endomyocardial fibrosis confirmed at histopathological examination (13). A viral-induced or immune-mediated endomyocardial injury with fibrosis repair, or a role of eosinophilic infiltration is suspected in some RCM cases (13). Additionally, necropsy descriptions suggested that LV endomyocardial fibrosis (corresponding to the pathological feature of RCM) and endomyocarditis (characterized by myocardial infiltrates predominantly of neutrophils and macrophages, in association with minimal myocytes degeneration) could be considered as temporally different phases of the same disease entity (13, 19). We can therefore hypothesize that some feline TRCM cases could possibly be related to endomyocarditis lesions resolving over time, similarly to what is suspected for feline TMT (4, 5).

The relationship between TRCM and TMT in cats remains to be elucidated. While both entities share several clinical features—such as a transient clinical course, the presence of a potential trigger or antecedent, suspected acute myocardial involvement, and predominant diastolic dysfunction—their echocardiographic phenotypes differ. These phenotypic differences raise the question of whether TRCM and TMT represent distinct clinical manifestations of a common underlying process, such as acute myocarditis, or rather reflect different temporal stages within the evolution of a single disease. However, histopathological data are lacking for both TRCM and TMT cats. Thus, the hypothesis of a shared inflammatory basis remains uncertain and warrants further investigation.

The origin of TMT is rarely identified. However, toxoplasmosis, bartonellosis, and sepsis secondary to infected wounds have been described in association with some TMT cases (5, 9). In our study, toxoplasmosis was only tested in one cat and was negative. Nevertheless, an infectious event was diagnosed in 4/17 TRCM cats (rhinitis, bronchopneumonia, feline leukemia and feline immunodeficiency viruses, and pyelonephritis). Additionally, in 4/17 TRCM cats including the two cats with bronchopneumonia and pyelonephritis, abnormalities compatible with an inflammatory process were identified at the complete blood count (i.e., neutrophilic leukocytosis in three cats, and eosinophilia in one).

In the present study, an antecedent event was identified in 65% of the TRCM cats, as also described in 33 to 71% of cats with TMT (4, 5). Similarly, in another study including cats with endomyocarditis identified at necropsy, a stressful antecedent event was reported in 75% of cats within 3 months before presentation to a veterinarian and within 2 weeks for 54% of cats including neutering, surgery, vaccination, cystitis, grooming, diarrhea, escape, and moving into a new home (19).

Markedly elevated blood concentration of cardiac troponin I has been reported in most feline TMT cases at presentation, with normalization during follow-up concomitantly with myocardial thickening resolution (4). In the present study, cardiac troponin I was available for two cats only. Nevertheless, the markedly elevated values at diagnosis followed by their normalization or sub-normalization at final examination, as described for TMT cats (4), is highly suggestive of a similar pathological process, involving acute myocarditis with myocardial lysis (4), although the time from diagnosis to resolution of echocardiographic lesions in TRCM cases appears to be longer than what has been reported in TMT cases, with a median of 156 days for the former compared to a median of 43 days to 3.3 months for the latter (4, 5).

According to our results, from a practical point of view the distinction between TRCM and NT-RCM cats at presentation appears to be difficult, since most clinical parameters were not significantly different between the two feline groups, with CHF being a highly frequent complication in all cases. Nevertheless, TRCM cats were significantly younger than cats with a primary RCM phenotype, as also described in feline TMT (median age of 2.0 years vs. 8 years for primary HCM phenotype) (4). Regarding echocardiographic variables, as described in cats with TMT (4), TRCM cats were characterized by a lower LA size than NT-RCM cats (as confirmed by significantly lower values of the LA:Ao ratio and the maximal LA diameter) and also by a lower RA diameter. However, discrimination between TRCM and NT-RCM cats cannot be made based on these imaging criteria because of marked overlap between the two groups.

There are currently few publications describing clinical outcomes and prognosis of cats with an RCM phenotype (14, 15, 20). In these studies, a relatively short life expectancy has been reported, with a median survival time comprised between 64 days for cats with respiratory distress and 667 days (14, 15, 20). In the present study, the median survival time for cats with NT-RCM (667 days) is similar to that reported on a larger feline population (i.e., 69 cats for which follow-up data > 24 h after diagnosis were available) (15). Due to the short life expectancy of NT-RCM cats, particularly those with CHF, the selection of NT-RCM cats was based on a relatively short follow-up period without normalization of cardiac remodeling. Therefore, we cannot exclude that some TRCM cats with a relatively slow cardiac recovery had been included in the NT-RCM group, which represents the main limitation of the present study. This study presents other limitations, mainly due to its retrospective nature. For NT-RCM cats, the RCM diagnosis was based on echocardiographic and Doppler criteria when available and was not confirmed by pathological examination of the heart. Additionally, owing to E and A wave fusion, Doppler evaluation of mitral valve inflow (which is one of the main imaging diagnostic tools used for RCM diagnosis in human patients) could be obtained for only 15 of the 33 cats included in the study, explaining that the RCM diagnosis was herein based on the echocardiographic criteria proposed by the ACVIM consensus statement on feline cardiomyopathies (1). Besides, time to evidence of echocardiographic resolution of the RCM phenotype may have been overestimated as owners of TRCM cats were not inclined to rapidly present their cats for a control visit when the latter became asymptomatic. Finally, no necropsy was performed on these cats, which could have permitted to confirm lesions of myocarditis or fibrosis.

## 5 Conclusion

In conclusion, this preliminary study describes another feline transient myocardial phenotype, characterized by CHF in all cats, most commonly occurring in young animals and with common reported antecedent events.

A definitive diagnosis of TRCM is only possible with follow-up. Nevertheless, clinicians should consider TRCM as a differential diagnosis from the first consultation, particularly in young cats presenting with an RCM phenotype and a clear antecedent event, and should be encouraged to measure cardiac troponin I. Close monitoring, including repeated echocardiographic evaluations and serial troponin I measurements over the weeks and months following the initial presentation, is recommended, particularly in cases where cardiac troponin I is elevated at diagnosis. Further prospective studies are needed on larger feline TRCM populations with systematic evaluation of cardiac troponin I at diagnosis and during follow-up, and with advanced evaluation of diastolic function, especially using tissue Doppler imaging in order to assess its ability to differentiate TRCM and NT-RCM cats at presentation.

Part of this study was presented as a poster at the 34^th^ annual European College of Veterinary Internal Medicine (ECVIM-CA) congress (Lyon, France).

## Data Availability

The raw data supporting the conclusions of this article will be made available by the authors, without undue reservation.

## Glossary

2D: two-dimensional
A: peak velocity of late diastolic transmitral flow
ACVIM: American College Veterinary Internal Medicine
Ao: aorta
CHF: congestive heart failure
E: peak velocity of early diastolic transmitral flow
E:A: ratio of E to A
HCM: hypertrophic cardiomyopathy
IVSd: interventricular septum at end-diastole
IVSs: interventricular septum at end-systole
LA: left atrium
LV: left ventricle
LVDd: left ventricular diameter at end-diastole
LVFWd: left ventricular free wall thickness at end-diastole
LVFWs: left ventricular free wall thickness at end-systole
LVDs: left ventricular diameter at end-systole
Max LAD: maximum left atrial diameter
NT-RCM: non transient restrictive cardiomyopathy
RCM: restrictive cardiomyopathy
SF%: shortening fraction (%)
TRCM: transient restrictive cardiomyopathy
TMT: transient myocardial thickening
